# Neuronal Ceroid Lipofuscinosis in a Domestic Cat Associated with a DNA Sequence Variant That Creates a Premature Stop Codon in *CLN6*

**DOI:** 10.1534/g3.120.401407

**Published:** 2020-06-09

**Authors:** Martin L. Katz, Reuben M. Buckley, Vanessa Biegen, Dennis P. O’Brien, Gayle C. Johnson, Wesley C. Warren, Leslie A. Lyons

**Affiliations:** * Neurodegenerative Diseases Research Laboratory and Department of Ophthalmology,; ^†^ Department of Veterinary Medicine and Surgery,; ^§^ Department of Veterinary Pathobiology,; ** Life Sciences Center, University of Missouri, Columbia, MO and; ^‡^ VCA Animal Specialty Group, San Diego, CA

**Keywords:** feline, whole exome sequencing, lysosomal storage disease, Batten disease, mutation, hereditary

## Abstract

A neutered male domestic medium-haired cat presented at a veterinary neurology clinic at 20 months of age due to progressive neurological signs that included visual impairment, focal myoclonus, and frequent severe generalized seizures that were refractory to treatment with phenobarbital. Magnetic resonance imaging revealed diffuse global brain atrophy. Due to the severity and frequency of its seizures, the cat was euthanized at 22 months of age. Microscopic examination of the cerebellum, cerebral cortex and brainstem revealed pronounced intracellular accumulations of autofluorescent storage material and inflammation in all 3 brain regions. Ultrastructural examination of the storage material indicated that it consisted almost completely of tightly-packed membrane-like material. The clinical signs and neuropathology strongly suggested that the cat suffered from a form of neuronal ceroid lipofuscinosis (NCL). Whole exome sequence analysis was performed on genomic DNA from the affected cat. Comparison of the sequence data to whole exome sequence data from 39 unaffected cats and whole genome sequence data from an additional 195 unaffected cats revealed a homozygous variant in *CLN6* that was unique to the affected cat. This variant was predicted to cause a stop gain in the transcript due to a guanine to adenine transition (ENSFCAT00000025909:c.668G > A; XM_003987007.5:c.668G > A) and was the sole loss of function variant detected. *CLN6* variants in other species, including humans, dogs, and sheep, are associated with the CLN6 form of NCL. Based on the affected cat’s clinical signs, neuropathology and molecular genetic analysis, we conclude that the cat’s disorder resulted from the loss of function of CLN6. This study is only the second to identify the molecular genetic basis of a feline NCL. Other cats exhibiting similar signs can now be screened for the *CLN6* variant. This could lead to establishment of a feline model of CLN6 disease that could be used in therapeutic intervention studies.

The neuronal ceroid lipofuscinoses (NCLs) are hereditary progressive neurological diseases that occur in humans and a number of animal species including dogs, cats, sheep, and cattle (Nakayama *et al.* 1993; Bildfell *et al.* 1995; Jolly 1995; Weissenböck and Rossel 1997; Houweling *et al.* 2006; Tammen *et al.* 2006; Frugier *et al.* 2008; Kuwamura *et al.* 2009; Mole *et al.* 2011; Chalkley *et al.* 2014; Katz *et al.* 2017). These disorders are characterized by apparently normal development followed by progressive declines in cognitive and motor functions, visual impairment, seizures, and in most cases culminate in premature death. A characteristic feature of the NCLs that distinguish them from most other inherited neurological diseases is massive accumulation of autofluorescent lysosomal storage bodies throughout the central nervous system as well as in other tissues and organs (Mole *et al.* 2011). The neurological signs and storage body accumulation are usually accompanied by progressive generalized brain atrophy.

NCL-causing sequence variants in humans have been identified in 13 genes. Different forms of NCL are currently designated as CLN1 through CLN14 based on the gene that harbors the disease-causing variant (the genetic bases of one form of NCL has yet to be identified) (Warrier *et al.* 2013). In dogs, different forms of NCL have been associated with sequence variants in the canine orthologs of eight of these genes (Katz *et al.* 2017). Sequence variants that cause NCL in sheep have also been identified in the ovine orthologs of three of the CLN genes (Tyynela *et al.* 2000; Tammen *et al.* 2006; Frugier *et al.* 2008). Although a number of cases of NCL have been reported in cats based on disease phenotype (Nakayama *et al.* 1993; Bildfell *et al.* 1995; Kuwamura *et al.* 2009; Chalkley *et al.* 2014), only one feline NCL-causing variant has been identified (a frameshift in *MFSD8* associated with CLN7 disease) (Guevar *et al.* 2019).

Whole genome sequencing (WGS) and whole exome sequencing (WES) are now feasible approaches for the interrogation of genomes for causal variants of diseases in domestic cats (Mauler *et al.* 2017; Guevar *et al.* 2019; Rodney *et al.* 2020). The associated costs of WGS and in particular, WES, are competitive with the time and resources costs associated with direct targeted Sanger sequencing of candidate genes. The V9.0 cat genome assembly has notable improvements due to the use of long-read sequences and an optical map for its construction (Buckley *et al.* 2020a). This strong cat assembly supported the development of an exome capture resource for use in domestic cat disease investigations (Rodney *et al.* 2020). Via the 99 Lives Cat Genome Project (http://felinegenetics.missouri.edu/99lives) a variant database of ∼200 cat genomes has been compiled (Buckley *et al.* 2020b). Utilization of this resource greatly facilitates the identification of potential causal variants for hereditary diseases (Lyons *et al.* 2016; Aberdein *et al.* 2017; Oh *et al.* 2017; Jaffey *et al.* 2019).

A domestic medium-haired cat exhibiting progressive neurological signs suggestive of NCL presented at a veterinary neurology clinic. Due to frequent intractable seizures, the cat was euthanized. To determine whether the cat suffered from a form of NCL, tissues were collected for histopathological evaluations and DNA was isolated for molecular genetic analysis. Whole exome sequencing was performed to identify candidate causal variants for the cat’s disease presentation.

## Materials And Methods

### Subject cat

A 20-month-old neutered male orange and white domestic medium haired cat presented at a veterinary neurology clinic due to frequent severe generalized seizures that were refractory to treatment with phenobarbital. The owner had acquired the proband and one of its littermates from a litter produced from a stray cat that delivered the kittens outside the owner’s home. Both littermates were kept indoors since weaning and received regular veterinary care, including standard vaccinations. The littermate of the affected cat has not exhibited any abnormal neurological signs. Due to the severity and frequency of its seizures, the proband was euthanized at 22 months of age.

### Clinical evaluations and necropsy

Prior to euthanasia the affected cat was examined by a veterinary neurologist (VB), who also interviewed the owner about the cat’s history. Magnetic resonance imaging of the cat’s brain was performed at the age of 20 months. From a urine sample obtained when the cat was 21.5 months old, quantitative urine organic acids analysis that can detect 76 different compounds potentially present in urine was performed in the Biochemical Genetics Laboratory at the University of California San Diego (UCSD) using gas chromatography-mass spectrometry, as previously described (Jones and Bennett 2010). Carnitine and acylcarnitine analyses were performed on plasma from a blood sample obtained at the same time as the urine sample. The latter analyses were performed by the UCSD Biochemical Genetics Laboratory using tandem mass spectrometry as previously described (Millington and Stevens 2011). These analyses identify the concentrations of free carnitine and the individual concentrations of 35 different acyl carnitines. In addition, the cat’s medical history dating to the age of 8 months was reviewed. Shortly after euthanasia, the cat’s brain was removed and placed in 10% buffered formalin. Pieces of liver, spleen, kidney, and muscle were also collected and frozen. The tissue samples were shipped to the University of Missouri for evaluation. All studies utilizing these tissues were approved by the University of Missouri Institutional Animal Care and Use Committee.

### Tissue morphological evaluation

Selected regions of the fixed brain were dissected, embedded in paraffin and sectioned. Sections were stained with periodic acid-Schiff reagent (PAS) and with Luxol fast blue (LFB) and examined by a veterinary pathologist (GCJ). Additional sections from the same blocks were immunostained for glial fibrillary acid factor (GFAP) and for ionized calcium-binding adapter molecule 1 (Iba1) (Schmutz *et al.* 2019; Villani *et al.* 2019). Adjacent slices of the same brain regions were washed in 0.17 M sodium cacodylate (pH 7.4), embedded in Tissue-Tek medium (Sakura Finetek, Torrance, CA), and frozen on dry ice. Sections of the frozen tissue were cut at a thickness of 8 μm with a cryostat, mounted on Superfrost Plus slides (Fisher Scientific, Pittsburgh, PA), and examined with fluorescence microscopy (Vuillemenot *et al.* 2011). Additional adjacent slices of the same brain regions were washed in the cacodylate buffer and then incubated at room temperature with constant gentle agitation for at least 24 hr in 2.5% glutaraldehyde in 0.1 M sodium cacodylate, pH 7.4. The samples were then post-fixed in osmium tetroxide and uranyl acetate and embedded in epoxy resin. Sections of these samples were cut at thicknesses of 70 to 90 nm, mounted in copper grids, and stained with uranyl acetate and lead citrate. These sections were examined with a JEOL JEM-1400 transmission electron microscope equipped with a Gatan digital camera.

### Molecular genetic analyses

Cat DNA samples were donated by the owners and archived in accordance with the University of Missouri Institutional Animal Care and Use Committee protocol study protocols 9056, 9178, and 9642. DNA was isolated from the frozen spleen sample by organic extraction and approximately 2.9 μg was submitted to the McDonnell Genome Institute at Washington University in St. Louis for whole exome sequencing. A domestic cat exome capture array (Roche Sequencing and Life Sciences, Wilmington, MA) for hybrid-capture target enrichment of the domestic cat exons was used to generate the sequence reads using Illumina sequencing technology (Buckley *et al.* 2020b). Automated dual indexed libraries were constructed with the KAPA HTP library prep kit (Roche) on the SciClone NGS platform (Perkin Elmer). Each library pool was hybridized with a custom Nimblegen probe set (Roche), targeting 35.9Mb of base space. An llumina NovaSeq6000 instrument was used to generate 150 bp paired end sequences to yield an average of 14 Gb of data per 35.9 Mb target exome, producing ∼60x genome coverage. Details of the whole exome sequencing and analyses have been previously reported for this case (Cat # 18 Fcat- 20617) (Buckley *et al.* 2020b). Sequences were aligned to cat genome assembly Felis_Catus_9.0 (GCA_000181335.4, BioProject: PRJNA16726) and processed using the methods outlined elsewhere (Buckley *et al.* 2020b). The variant call file was combined with variants determined from 40 additional cats with WES data. The variant data were annotated, visualized and filtered using VarSeq (GoldenHelix, Inc., Bozeman, MT). Annotations were obtained from Ensembl release 98. The variant data were analyzed by assuming the affected cat was homozygous for the causal variant and that no other cat in the dataset had the variant present. A candidate *CLN6* disease variant in the affected cat was validated by direct Sanger sequencing. PCR primers were designed using Primer3 to flank the variant the *CLN6* variant (B3:39334330; c.668G > A; p.Trp223Ter) (Forward primer: 5′- CCTTACACGAGGAGCTGAGG -3′ and Reverse primer: 5′- CTACACAGGGGAGGAAGCAG -3′) using cat sequence ENSFCAG00000025647 for *CLN6*. The amplicon size was 468 bp (B3:39333980-39334447) and was 60% GC-rich. PCR was conducted in a 25µL reaction, using 1U AccuPrime GC-Rich DNA Polymerase (Thermo Fisher Scientific, Waltham, MA), 1x AccuPrime GC-Rich Buffer A, 0.4 µM each primer, 30 ng gDNA. PCR conditions were an initial denaturation at 96° for 3:00min, followed by 35 cycles of 96° for 30s, 58° for 30s, 70° for 30s with a final extension at 70° for 10:00 min. The amplicon was visualized on a 1% agarose gel via electrophoresis at 90V for 75 min and ethidium bromide staining. The amplicon was gel extracted using an QIAquick Gel Extraction Kit (Qiagen, Hilden, Germany) and eluted in 50 µL TE. Sanger sequencing was conducted at the MU DNA Core Facility using an Applied Biosystems 3730xl DNA Analyzer (Applied Biosystems, Foster city, CA, USA) with BigDye Terminator v3.1 Cycle Sequencing Kit (Applied Biosystems). DNA sequences were visualized using the software Sequencer (GeneCodes Corp., Ann Arbor, MA, USA). The identified variants were also examined in the 99 Lives dataset that includes WGS data from ∼195 cats (Jaffey *et al.* 2019; Buckley *et al.* 2020a), also using VarSeq software.

### Data availability

All of the sequence variants unique to the affected cat and that had read depths >13 are included in Supplemental Table 1. Exome sequencing data are available at the Sequence Read Archive under accession number PRJNA627536. The sequence for the 195-cat analysis of the 99 Lives cat genome sequencing project (Buckley *et al.* 2020a) are submitted to the NCBI short read archive under BioProject: PRJNA308208, PRJNA288177. Supplemental material available at figshare: https://doi.org/10.25387/g3.12448676.

## Results

### Clinical and neurological findings

**Figure 1:**
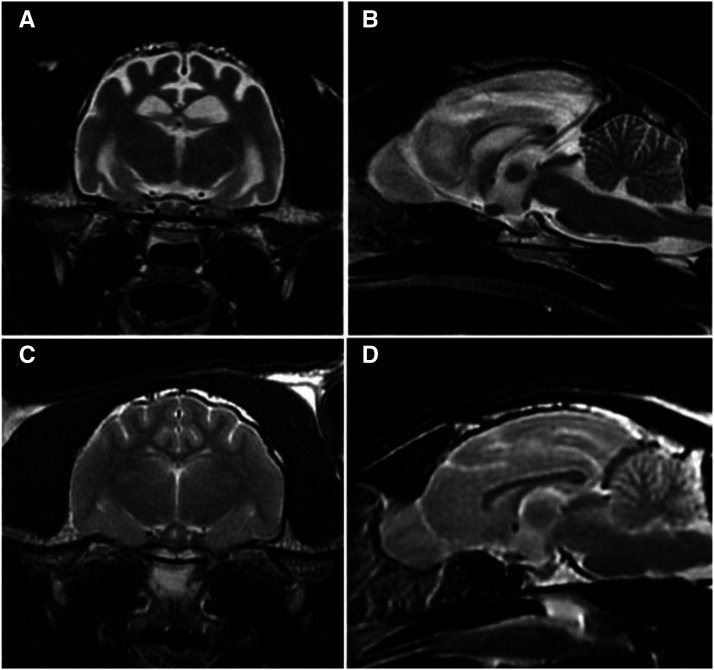
Axial (A) and sagittal (B) T2-weighted magnetic resonance images of the affected cat’s brain showing loss of gray/white matter distinction in the cerebral cortex and diffuse cerebral cortical atrophy (B). Comparable axial (C) and sagittal (D) images of the brain of a healthy young adult cat.

The subject male cat appeared neurologically healthy until approximately 19 months of age, at which time it had a generalized tonic-clonic seizure followed by a second similar seizure about 1 month later. The latter seizure was characterized by convulsions that lasted approximately 1 min. For the next 3-5 min the cat was disoriented, unsure, and stumbling after which it was seemingly back to normal. Another similar seizure occurred about one week later, at which time phenobarbital administration was initiated. Within 2 weeks the cat was exhibiting myoclonus of the face and head. The myoclonic episodes occurred 2 to 3 times per day and were refractory to phenobarbital treatment. His menace response was absent in both eyes and his vision appeared to be impaired. No metabolic, infectious or toxic cause for the seizures was identified. Despite therapy with several antiepileptic drugs, daily seizures, including episodes of abnormal behavior with facial myoclonus, continued to occur and the visual impairment did not resolve. Upon neurological examination at 20 months of age, the cat was ataxic, mildly disoriented, and his mentation appeared to be dull. The cat was lethargic, although it was not clear if this was due to progression of the disease or to side effects of the antiepileptic medication. Magnetic resonance imaging performed at 21 months of age showed diffuse brain atrophy with loss of gray/white matter distinction on T2 weighted images (Figure 1). Due to the progression of signs and poor response to therapy, the cat was euthanized at 22 months of age and a necropsy performed.

Analysis of a urine sample did not identify any compounds that were present in concentrations significantly outside the reference range for normal adult cats. Plasma free carnitine and acetyl, propionyl, and octenoyl carnitines in the affected cat were below the reference range for healthy cats (Jacobs *et al.* 1990). Because free and total acyl carnitine levels have been reported to differ significantly between male and female cats (Jacobs *et al.* 1990), these levels in the affected cat are compared to those previously reported for healthy adult male cats only (Table 1). Free, acylated and total carnitine levels in the affected cat are all below the mean levels reported for healthy adult male cats (Jacobs *et al.* 1990).

**Table 1:** Plasma carnitine levels in the affected cat and healthy adult male cats

Carnitine Form	Affected Cat	Healthy Male Cats*^a^*
Free (μM)	9.14	11.1 ± 3.6
Acylated (μM)	1.57	2.6 ± 1.6
Total (μM)	10.71	13.6 ± 4.3
% Free	85	82

a Mean ± SD for 9 healthy adult male cats (Jacobs *et al.* 1990)

### Pathology findings

**Figure 2:**
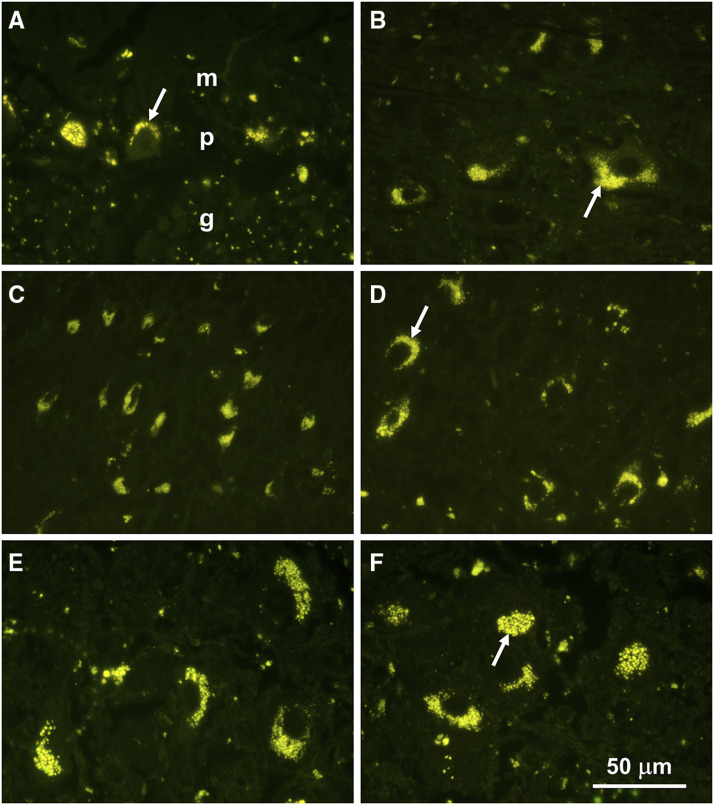
Fluorescence micrographs of sections of the cerebellar cortex (A), a deep cerebellar nucleus (B), the parietal cerebral cortex (C and D), and brainstem nuclei (E and F). In the cerebellar cortex, the largest accumulations of storage bodies were present in the Purkinje cell layer (p), with scattered storage body aggregates in the granule cell (g) and molecular (m) layers.

Fluorescence microscopy of unstained cryostat sections revealed substantial accumulations of autofluorescent storage bodies in all areas of the brain that were examined (Figure 2). This storage material exhibited a yellow emission when stimulated by blue light that is characteristic of the storage material in the NCLs (Siegismund *et al.* 1982; Katz and Gerhardt 1990; Savill *et al.* 1995; Kuwamura *et al.* 2003; Cannelli *et al.* 2009; Mole *et al.* 2011; Vuillemenot *et al.* 2011). Accumulations of the storage bodies in most neurons were perinuclear with aggregates usually concentrated at one pole of the cell.

**Figure 3:**
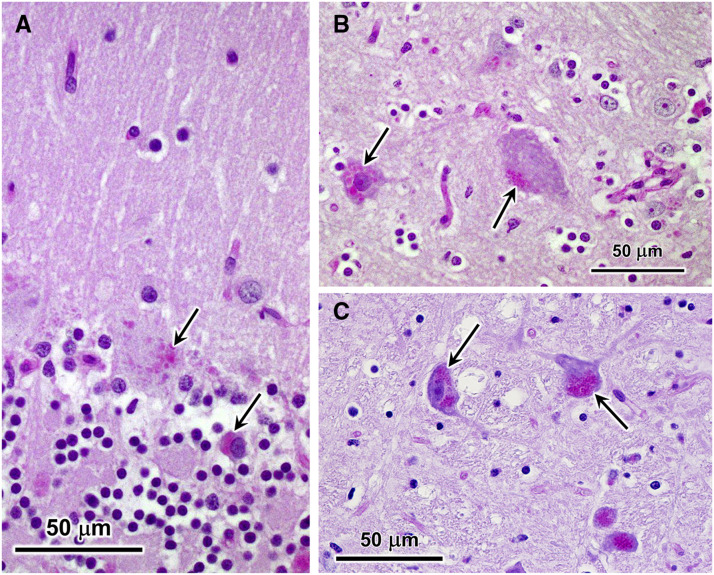
Light micrographs of sections of cerebellar cortex (A), cerebral cortex (B), and brainstem (C) from the affected cat. Sections from the cerebellar cortex and cerebral cortex were stained with periodic acid-Schiff (PAS) reagent, and the section from the brainstem was stained with a combination of PAS and Luxol fast blue (LFB). Purkinje cells (top arrow in A) and occasional cells in the molecular layer (lower arrow in A) contained aggregates of PAS-stained inclusions. Similar inclusions were present in cerebral cortical neurons (arrows in B). In brainstem neurons, similar inclusions appeared purple when stained with a combination of PAS and LFB (arrows in C).

**Figure 4:**
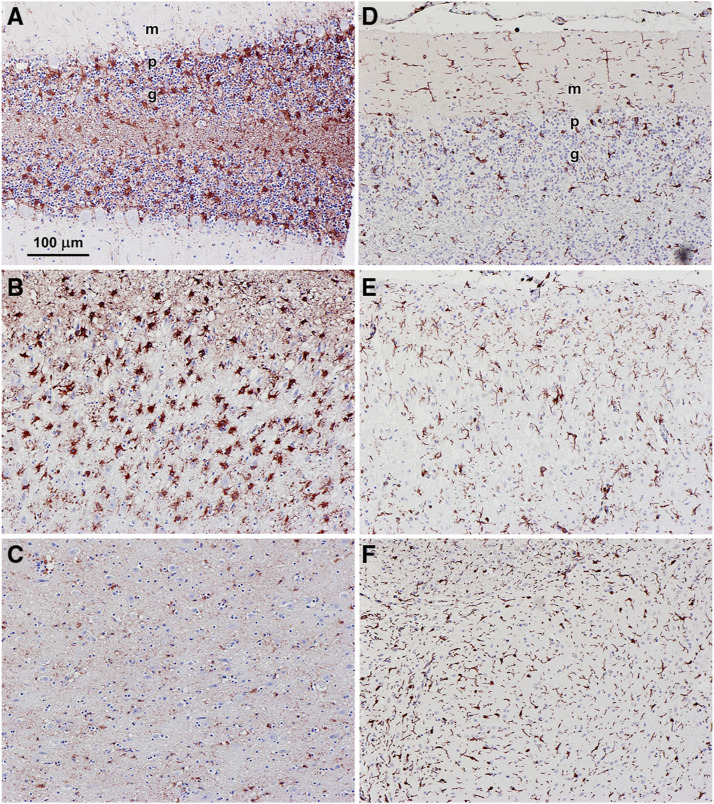
Light micrographs of sections of cerebellar cortex (A and D), cerebral cortex (B and E), and brainstem (E and F) from the affected cat immunostained for GFAP (A-C) and Iba1 (D-F). GFAP immunostaining demonstrates activated astrocytes and Iba1 immunostaining indicates activated microglia, both of which are indicative of neuroinflammation. Bar in (A) indicates magnification of all 6 micrographs. Layers of the cerebellar cortex indicated in (A) and (D) – m: molecular layer; p:Purkinje cell layer; g: granule cell layer.

The inclusions observed by fluorescence microscopy in various brain regions of the affected cat stained with PAS reagent and with LFB (Figure 3). PAS stains sugars with adjacent hydroxyl groups, and in this case, what are stained are most likely glycoproteins and/or oligosaccharyl dolichols (Wisniewski *et al.* 1988; Elleder 1989; Elleder *et al.* 1989; Wisniewski and Maslinska 1990). LFB has been shown to stain the storage bodies that accumulate in cells in the NCLs (Cho *et al.* 1986; Weissenböck and Rossel 1997; Ranta *et al.* 2001; Sinha *et al.* 2004). The granule cell layer of the cerebellum was moderately depleted of cells and the cerebral cortex was thinner than normal. Astrocyte activation based on immunostaining with an anti-GFAP antibody was present in the cerebellar cortex and cerebral cortex, but not in the brainstem (Figure 4 A-C). In the cerebellar cortex, activated astrocytes were present primarily in the Purkinje and granule cell layers. Activated astrocytes were present in all layers of the cerebral cortex. Immunostaining with an anti-Iba1 antibody revealed abundant activated microglia in all three brain regions (Figure 4 D-F). In the cerebellar cortex, activated microglia were present primarily in the molecular and granule cell layers (Figure 4D). Activated microglia were present throughout the cerebral cortex and brainstem Figure 4 E and F).

**Figure 5:**
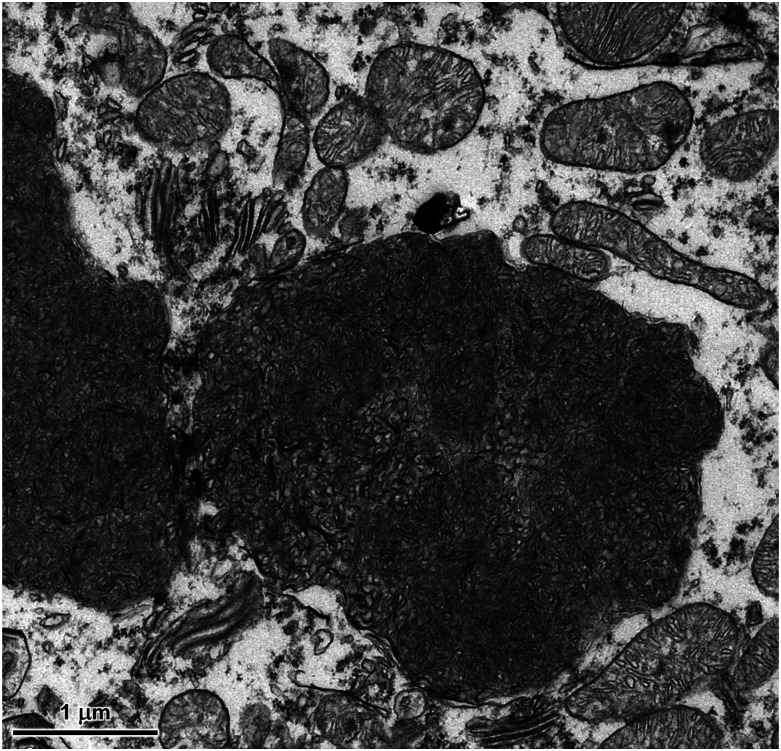
Electron micrograph of disease-specific storage body in a cerebellar Purkinje cell of the affected cat. Ultrastructurally similar storage bodies were present in cells of the molecular and granule cell layers of the cerebellar cortex.

**Figure 6:**
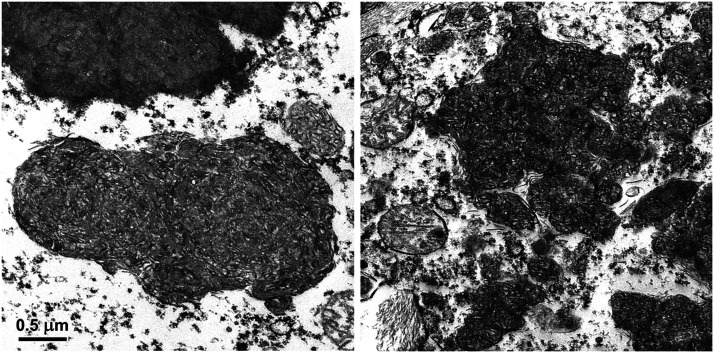
Electron micrographs of disease-specific storage bodies in a cerebral cortical neuron (left) and in a neuron in the brainstem (right) of the affected cat. Bar on left indicates magnification of both micrographs.

At the ultrastructural level, the contents of the storage bodies consisted almost exclusively of tightly-packed membrane-like structures that were both vesicular and linear in cross-section (Figures 5 and 6). The organization of these structures within the storage bodies appeared to be random. Within each area of the brain, there was some variability in the organization of these membrane-like inclusions between different storage bodies. This heterogeneity was similar for all 3 brain regions examined.

### Molecular genetic findings

**Figure 7:**
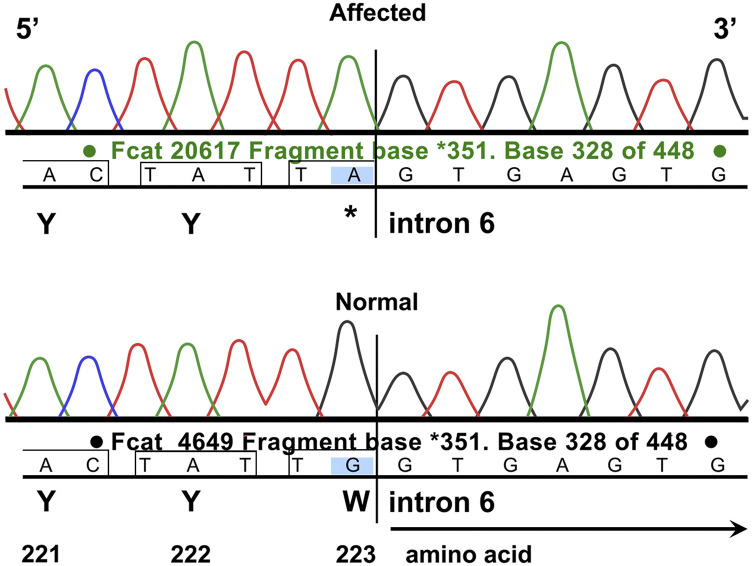
Direct Sanger sequencing validation of the *CLN6* c.668A variant in the affected cat. Electropherograms of the ENSFCAT00000025909:c.668G > A; XM_003987007.5:c.668G > A and the flanking nucleotides in *CLN6*. The affected cat Fcat – 20617 (top trace) is homozygous for the adenine variant, and the control cat Fcat - 4649 (bottom trace) is homozygous wildtype for guanine. Sequence presented as positive strand reading 5′ – 3′. The variant likely disrupts the slice donor site of exon 6, allowing read-through and causing the p.Trp223ter. If splicing is intact, the same termination codon will occur (the third nucleotide of codon 223 (G) is the first nucleotide of exon 7).

The WES dataset used for analysis includes unaffected domestic cats (Buckley *et al.* 2020b; Rodney *et al.* 2020). The depth of exon coverage for the proband cat averaged 94.2x. The affected cat was homozygous for 109 variants that were unique within the WES dataset (Supplemental Table1). The read depth was <15 for 36 variants, which were not further considered as a standard for heterozygous calling is generally considered 15 - 20x coverage. All remaining variants in the affected cat (n = 73) had read depths ranging from 15 to 389 (Supplemental Table 1). These variants were called in all cats in the WES dataset. The genotype qualities (GQ) for the variants in the proband ranged from 45 – 99. All but four variants had a GQ equal to 99. The effects on the transcripts included 15 variants that were associated with splice regions but not the canonical splice donor/acceptor sites, 55 variants were predicted to result in a single amino acid change in the encoded protein relative to the reference sequence, two variants were predicted as in-frame deletion or insertions and one variant was predicted as a 5′ UTR premature start codon gain. A variant was identified in *CLN6* that was predicted to cause a stop gain in the transcript by a guanine to adenine transition (ENSFCAT00000025909:c.668G > A; XM_003987007.5:c.668G > A) and was the sole loss of function variant detected. The GQ of the variant call was the highest possible of 99. The single nucleotide polymorphism is predicted to cause a tryptophan amino acid change to an termination codon (p.Trp223Ter) approximately 43–71% into the transcript, depending of the transcript used for annotation (ENSFCAT00000025909,ENSFCAT00000058092,ENSFCAT00000062783,ENSFCAT00000055735). Cat *CLN6* (XM_003987007.5) codes from the positive strand, whereas *CLN6* codes from the negative strand in humans (NM_017882.3). The cat c.668G > A variant is at the exon 6 intron exon boundary. Alignment of the cat sequence to human indicates conservation of the splice junction at exons 6 and 7, (Figure 7), suggesting the last two nucleotides of exon 6 and the first nucleotide of exon 7 code for the expected tryptophan codon (*i.e.*, splicing frame 2) (Shapiro and Senapathy 1987). Considering this conservation, the c.668G > A actually disrupts the canonical splice donor site at the 3′ of exon 6 TG|gt, converting the junction to TA|gt. If the disruption allows read through, the p.Trp223Ter occurs, creating an amber stop codon. If normal splicing occurs, the same termination codon is produced.

The frequencies of the 74 variant alleles in the 99 Lives WGS dataset that includes ∼195 cats were 0.00–12.1% (Supplemental Table 1). The *CLN6* c.668A variant allele was unique to the proband. Nineteen variant alleles were absent in the 99 Lives dataset, and were thus unique to the case. These variant alleles include two in-frame insertion/deletions, three splice region variants, and 13 missense variants, representing 15 known genes and four cat-specific undefined transcripts. Twenty-eight variant alleles had an alternate allele frequency <1% and are considered rare, while 26 variant alleles had alternate allele frequencies >1% and are considered polymorphic in cats.

*CLN6* variants were identified in the ∼195 cats of the 99 Lives cat whole genome sequencing project (Jaffey *et al.* 2019) (Table 2). Sixteen missense, synonymous or splice region variants were identified in the WGS 99 Lives data of 195 cats, however the loss of function variant (alternative) allele identified in the proband was not present. Two missense, one splice region and one synonymous variant are suggested as likely benign in the 99 Lives cats based on relatively high alternative allele frequencies (> 0.01). The same variant alleles are also present in the exome data (Table 3). No cats represented in the WGS or WES reference databases were reported to have exhibited neurological signs characteristic of NCL-like disorders. The *CLN6* c.668A variant was confirmed as homozygous in the proband by direct Sanger sequencing (Figure 7).

**Table 2:** ***CLN6* variants (transcript ENSFCAT00000025909) in cat whole genome sequences – 99 Lives***^a^*

Chr:Pos	Ref/Alt	Allele Counts	# Alleles	Allele Freq.	# Het	# Homo	Effect	Coding Change	Protein Change
B3:39320853	C/G	33	390	0.085	27	3	missense	c.71C > G	p.Ser24Cys
B3:39327181	G/A	49	390	0.126	23	13	missense	c.118G > A	p.Gly40Ser
B3:39331723	C/T	175	390	0.449	81	47	splice region	c.202-6C > T	———
B3:39331755	G/A	11	390	0.028	9	1	synonymous	c.228G > A	p.Pro76=
B3:39331770	C/T	1	390	0.003	1	0	synonymous	c.243C > T	p.Ser81=
B3:39331773	G/T	1	390	0.003	1	0	synonymous	c.246G > T	p.Val82=
B3:39331797	C/T	1	390	0.003	1	0	synonymous	c.270C > T	p.Tyr90=
B3:39331801	A/G	2	390	0.005	2	0	missense	c.274A > G	p.Ile92Val
B3:39331815	C/T	2	390	0.005	2	0	synonymous	c.288C > T	p.Phe96=
B3:39331818	T/C	2	390	0.005	2	0	synonymous	c.291T > C	p.Leu97=
B3:39332915	G/C	1	390	0.003	1	0	missense	c.339G > C	p.Met113Ile
B3:39332917	T/C	1	390	0.003	1	0	missense	c.341T > C	p.Ile114Thr
B3:39332918	C/G	1	390	0.003	1	0	missense	c.342C > G	p.Ile114Met
B3:39332935	C/T	1	390	0.003	1	0	missense	c.359C > T	p.Thr120Ile
B3:39332940	G/A	1	390	0.003	1	0	missense	c.364G > A	p.Val122Ile
B3:39336061	A/C	2	390	0.005	2	0	missense	c.794A > C	p.Tyr265Ser

a Data from 99 Lives Cat whole genome sequences annotated with Ensembl 97 (Jaffey *et al.* 2019).

**Table 3:** All *CLN6* variants in 41 cat exome sequences (including the affected cat).

Chr:Pos	Ref/Alt	Allele Counts	Allele Freq.	# Alleles	# Het	# Homo	Effect	Coding Change	Protein Change
B3:39320827	A/C	1	0.0125	80	1	0	synonymous	c.45A > C	p.Gly15=
B3:39320845	G/C	1	0.0125	80	1	0	synonymous	c.63G > C	p.Pro21=
B3:39320853	C/G	4	0.05	80	4	0	missense	c.71C > G	p.Ser24Cys
B3:39327136	T/C	38	0.475	80	14	12	intron	c.87-14T > C	________
B3:39327181	G/A	14	0.175	80	6	4	missense	c.118G > A	p.Gly40Ser
B3:39331721	C/T	1	0.0125	80	1	0	splice region	c.202-8C > T	________
B3:39331723	C/T	31	0.3875	80	13	9	splice region	c.202-6C > T	________
B3:39331755	G/A	2	0.025	80	2	0	missense	c.228G > A	p.Pro76=
B3:39331797	C/T	1	0.0125	80	1	0	missense	c.270C > T	p.Tyr90=
B3:39331818	T/C	1	0.0125	80	1	0	missense	c.291T > C	p.Leu97=
B3:39331834	C/T	1	0.0125	80	1	0	splice region	c.300+7C > T	________
B3:39332882	T/C	1	0.0125	80	1	0	synonymous	c.306T > C	p.Ile102=
B3:39333074	G/A	1	0.0125	80	1	0	intron	c.489+9G > A	________
B3:39334192	C/G	1	0.0125	80	1	0	intron	c.546-16C > G	________
B3:39334193	A/G	1	0.0125	80	1	0	intron	c.546-15A > G	________
B3:39334201	G/A	1	0.0125	80	1	0	splice region	c.546-7G > A	________
B3:39334330*^a^*	G/A	2	0.025	80	0	1	stop gained	c.668G > A	p.Trp223Ter
B3:39335924	C/T	1	0.0125	80	1	0	intron	c.669-12C > T	________
B3:39335948	C/G	1	0.0125	80	1	0	synonymous	c.681C > G	p.Thr227=
B3:39336199	G/A	1	0.0125	80	1	0	missense	c.932G > A	p.Arg311Gln

a Alternative allele variant present only in the proband.

## Discussion

The clinical signs exhibited by the subject cat, along with the histological and electron microscopic findings, established that this animal suffered from a form of NCL. Sequence variants in at least 13 genes have been associated with different forms of NCL, designated CLN1 – CLN12 diseases (Warrier *et al.* 2013). There is a significant degree of overlap in disease signs, histopathology, and storage body ultrastructure between the different NCLs. However, some of the NCLs can be clearly distinguished from other forms based on age of onset and rate of clinical disease progression and the ultrastructural appearance of the lysosomal storage material. For example, CLN2 disease is characterized by disease onset and progression to end-stage disease early in the normal lifespan and a unique ultrastructural appearance of the lysosomal storage material (Mole *et al*., 2005). Based on the disease phenotype of the affected cat, it appeared unlikely that it suffered from any of the CLN1, CLN2, CLN4, or CLN12 forms NCL. In order to determine which of the remaining forms of NCL the subject cat had, it was necessary to perform molecular genetic analyses.

To identify DNA sequence variants that might be associated NCL in the affected cat, a new cat whole exome capture resource for WES was employed. The targeted WES leads to more rapid computation times and less use of computational resources, significantly lowering the cost for implementing precision/genomic medicine for domestic cats and for identification of potential feline models for human inherited disorders. Rapid workflows and quick access to sequencing instrumentation allows the potential for a genetic diagnosis of a rare disease to be conducted within a timeframe that could allow more personalized and targeted treatment to the patient early in the disease progression. As with genome approaches in human medicine, the success of the WES approach for identifying disease-associated sequence variants is highly dependent on the heritability and frequency of the disease, the completeness of the exome capture resource, and the datasets available for comparison. In this case, most NCLs have clear, autosomal recessive modes of inheritance and these diseases appear to be rare in cats. The new long-read assembly of the domestic cat genome is one of the most complete assemblies in animals. The collaborative efforts of the cat genetics community have produced the WGS 99 Lives dataset that complimented the exome data evaluations in this study and that has also facilitated identification of sequence variants associated with other feline hereditary disorders.

Seventy-four variants were unique and homozygous in the WES of the NCL cat. However, inspection of the allele frequencies of the same variants in the 99 Lives WGS dataset identified 55 variants in other cats and thus not unique to the NCL cat. Therefore, these variants were eliminated as likely candidate variants. Although a causal variant could potentially be present in another cat or cats in the reference data sets, probably in the heterozygous state, the likelihood of sampling a second cat with the candidate variant by random chance is extremely low, particularly since no other cats in the 99 Lives dataset are reported as potentially having an NCL-like condition. None of the remaining 18 variants affect known NCL candidate genes. The variant suggested to cause NCL in the affected cat should produce a termination codon in the coding of the CLN6 protein. This variant was homozygous and unique in the NCL cat in the exome data and was not present in the 99 Lives WGS dataset of 195 cats. The variant disruption is found in exon 2 - 6 depending on the *CLN6* transcript, suggesting loss of the 25–60% of the 3′ portion of the protein.

A number of cases of NCL have been reported in domestic cats based on clinical signs and pathology (Nakayama *et al.* 1993; Bildfell *et al.* 1995; Weissenböck and Rossel 1997; Kuwamura *et al.* 2009; Furusawa *et al.* 2012; Chalkley *et al.* 2014), but this is only the second case of feline NCL in which the underlying molecular genetic cause has been determined. In one previously reported case of NCL in a domestic short-haired cat, no candidate disease sequence variants were detected by targeted sequencing of the NCL genes *PPT1*, *CLN3*, *CLN5*, *CLN8* or *CTSD* (Chalkley *et al.* 2014). In another unrelated cat with NCL, no potential deleterious variants were found in *CLN3* (Furusawa *et al.* 2012). These previous studies illustrate the limitations of sequencing individual candidate genes compared to WGS/WES approaches for identification of disease-associated variants. The only other pathogenic variants responsible for feline NCL were identified in *MFSD8* of a domestic shorthaired cat using WGS (Guevar *et al.* 2019). *MFSD8* variants underlie the CLN7 form of NCL in human subjects (Mole *et al.* 2011) and dogs (Katz *et al.* 2017). Therefore, NCLs in domestic cats result from deleterious variants in at least two of the known NCL genes. The same disease variant discovered in *CLN6* here may have been present in at least some of the other previously-reported cases that were not subjected to WES or WGS analyses. This *CLN6* variant can now be screened for in cats showing signs of NCL and in their close relatives. Such screening will facilitate determination of how prevalent this variant is among affected cats and can be used to inform breeding decisions. In those cats with phenotypically confirmed NCL in which neither the NCL-associated *CLN6* or *CLN7* variant is present, WES analysis is an efficient approach for identifying other causal variants in the feline orthologs of known NCL genes.

Many neurodegenerative diseases, including the NCLs, are characterized by neuroinflammation as indicated by astrocyte and microglial activation (Kay and Palmer 2013; Macauley *et al.* 2014; Shyng and Sands 2014; Drack *et al.* 2015; Groh *et al.* 2017; Degan *et al.* 2018; Liberman *et al.* 2018; Niranjan 2018). These indicators of inflammation were present in the regions of the brain that were examined in the proband cat. It has been hypothesized that NCL-associated neuroinflammation contributes to the disease pathogenesis (Qiao *et al.* 2007; Groh *et al.* 2013; Kay and Palmer 2013; Xiong and Kielian 2013; Groh *et al.* 2017). However, anti-inflammatory drug treatments targeted to the CNS have produced variable results in attenuating NCL disease progression (Seehafer *et al.* 2011; Kay and Palmer 2013; Augustine and Mink 2016; Groh *et al.* 2017; Dannhausen *et al.* 2018). Developing a better understanding of the molecular mechanisms that underlie neuroinflammation associated with the NCLs will facilitate development of rational approaches to better targeted anti-inflammatory treatments that can be tested in animal models such as the CLN6 cat.

In human and animal CLN6 disease, and in other forms of NCL, a major component of the storage material is the small subunit c protein of the inner mitochondrial membrane protein ATP synthase (Palmer *et al.* 1989; Fearnley *et al.* 1990; Ansari *et al.* 1995; Kominami *et al.* 1995; Tyynelä *et al.* 1997; Umehara *et al.* 1997; Jolly and Walkley 1999; Ranta *et al.* 2001; Cook *et al.* 2002; Cao *et al.* 2011; Palmer *et al.* 2013). This protein contains a trimethyl-lysine (TML) residue (Katz *et al.* 1994; Katz *et al.* 1995; Katz 1996; Katz *et al.* 1997; Chen *et al.* 2004). TML is a precursor in the biosynthesis of carnitine, which plays an important role in fatty acid metabolism (Katz 1996; Adeva-Andany *et al.* 2019). In previous studies, plasma carnitine levels have been found to be low in dogs with the CLN8 form of NCL and in children with the CLN3 form of NCL (Katz and Siakotos 1995; Katz 1996). That feline CLN6 disease is associated with below average plasma carnitine levels is consistent with the hypothesis that mitochondrial subunit c protein turnover is a major source of the TML precursor of carnitine, suggesting plasma carnitine levels in children with CLN6 disease should be assessed and may have implications for treating the disease. However, plasma carnitine levels in the affected cat, although below average, were within the reference range for male cats. Since blood carnitine levels are dependent on both dietary intake and endogenous biosynthesis,it will be necessary to control for dietary intake in affected and control cats to determine whether endogenous biosynthesis of carnitine is altered in feline CLN6 disease.

The ultrastructure of the storage bodies in the affected cat is quite similar to that of the storage bodies in the brains of dog, sheep and of human subjects with the CLN6 form of NCL (Mole *et al.* 2005; Katz *et al.* 2011; Mole *et al.* 2011). The storage body ultrastructure is quite distinctive for some forms of NCL, particularly CLN2 disease. However, the ultrastructural appearance of the storage bodies in CLN6 disease is similar to that of other forms of NCL, particularly CLN5 disease (Gilliam *et al.* 2015; Kolicheski *et al.* 2016; Villani *et al.* 2019). Thus, although the storage body ultrastructure can be useful in confirming a molecular genetic diagnosis, ultrastructure alone cannot distinguish CLN6 disease from some of the other NCLs.

The success of WES and WGS analyses in identifying the molecular genetic bases of two feline NCLs should open the door to the discovery of other sequence variants that cause NCL in domestic cats. In the past, the only way to determine whether a cat with progressive neurological signs was suffering from a form of NCL was to examine neural tissues collected at necropsy. As a result, most cases of feline NCL have likely gone unreported. Genetic testing can now be used to easily screen for the *CLN6* disease variant identified in the proband in this study as well as for the *MFSD8* variants identified in another domestic cat with putative CLN7 disease. Targeted screening may result in the identification of additional cats with these variants as well as symptomatic cats that do not harbor these NCL variants. Cats in the latter category would be candidates for WES or WGS analysis.
